# Combination of sibutramine and topiramate for the treatment of obesity: the SIBAMATE retrospective cohort study: Sibutramine and topiramate for the treatment of obesity

**DOI:** 10.1186/s13098-025-01842-1

**Published:** 2025-07-21

**Authors:** Cintia Cercato, Matheo A. M. Stumpf, Gabriel Neimann da Cunha Freire, Eduardo Zanatta Kawahara, Ariana E. Fernandes, Maria E. de Melo, Marcio C. Mancini

**Affiliations:** Obesity Unit, Division of Endocrinology and Metabolism, University of São Paulo Medical School Hospital, Av. Dr. Enéas Carvalho de Aguiar, 155 8° andar, Bloco 8ª, Cerqueira César, São Paulo, CEP 05403-000 Brazil

**Keywords:** Obesity, Sibutramine, Topiramate, Antiobesity medication, Weight loss

## Abstract

Long-term treatment of obesity with lifestyle changes alone is unsustainable for most individuals. Antiobesity medications are recommended for individuals with a body mass index (BMI) ≥ 30 kg/m² or ≥ 27 kg/m² with one or more comorbidities. In Brazil, the prescription of combined sibutramine plus topiramate for obesity management is common in daily clinical practice. However, data on its effectiveness and safety are lacking. Thus, the objective of this study was to evaluate this combination for treating obesity in a real-world scenario. Methods: This retrospective cohort study included individuals with obesity ≥ 18 years prescribed sibutramine and topiramate for at least 3 months between 2012 and 2022 at a large tertiary healthcare center. Baseline and follow-up data were collected from medical records. Results: Among 861 medical records screened, 246 (28.6%) were included. Most participants were female (86.2%) with a mean age of 42.8 ± 12.7 years, 52% had hypertension, 31.3% type 2 diabetes and 30% dyslipidemia. The average baseline BMI and weight were 39.7 kg/m² and 104.2 kg, respectively. The mean daily doses of sibutramine and topiramate were 11 ± 2.1 mg and 119.7 ± 54.7 mg, respectively. There was a significant change in body weight precociously at 3 months on the combination (96.8 *±* 20.7 kg, *p* < 0.001), with 61.8% of patients achieving at least *≥* 5% of weight loss, 29.4% *≥*10% and 10.9% *≥*15%. At 36 months, 64% maintained at least *≥* 5% of weight loss, 40.6% *≥*10% and 26.5% *≥*15%. Common adverse effects included paresthesia, memory impairment, bradyphrenia and elevated blood pressure. The discontinuation rate was 24.4%. No major safety concern was observed in a mean follow-up of 25.3 months. Conclusion: In a real-world setting, sibutramine and topiramate combination therapy was associated with clinically meaningful weight loss alongside a good tolerability profile.

## Introduction

Obesity is a chronic condition and a global public health concern. It is projected that the number of adults living with obesity will rise from 0.81 billion in 2020 to 1.53 billion in 2035 [1].

Although lifestyle changes should be provided to all individuals with obesity, their effectiveness as a standalone intervention is modest [2]. This is largely related to low compliance in a long-term intervention [3], leading to weight regain and frustration. Consequently, in this scenario, antiobesity drug therapy plays a crucial role as an adjuvant for weight loss [4].

The current decade has witnessed a significant transformation in obesity pharmacotherapy, with the emergence of new medications offering not only cardiovascular safety but also cardiovascular protection​ [5]. The potency of achieving a weight loss greater than 10–15% is also noticeable [6–8]. However, access to these drugs remains limited due to high costs, creating a substantial barrier, particularly in low- and middle-income countries where 79% of adults with overweight or obesity are expected to reside by 2035 [1].

This context underscores the urgent need for affordable antiobesity medications. While the phentermine-topiramate combination is widely used in the United States, with a total weight loss ranging from 8 to 10 kg depending on the dosage [9, 10], it is unavailable in Brazil. Alternatively, the off-label combination of sibutramine and topiramate is commonly used in Brazilian daily clinical practice due to its low price, with some similarities in the mechanism of action of phentermine plus topiramate [11].

Nevertheless, to date, no trials evaluated the effectiveness in weight loss or safety profile of the sibutramine-topiramate combination. Thus, the aim of this study is to assess this combination therapy in a real-world clinical setting, providing crucial insights into its potential role in obesity management.

## Methods

The SIBAMATE retrospective cohort study was conducted at a single academic tertiary care center with extensive experience in obesity management. The inclusion criteria consisted of patients aged *≥* 18 years with body mass index (BMI) ≥ 30 kg/m² or BMI *≥* 27 kg/m² with at least one comorbidity (type 2 diabetes, hypertension or dyslipidemia), who received sibutramine plus topiramate for at least 3 months.

**Fig. 1:**
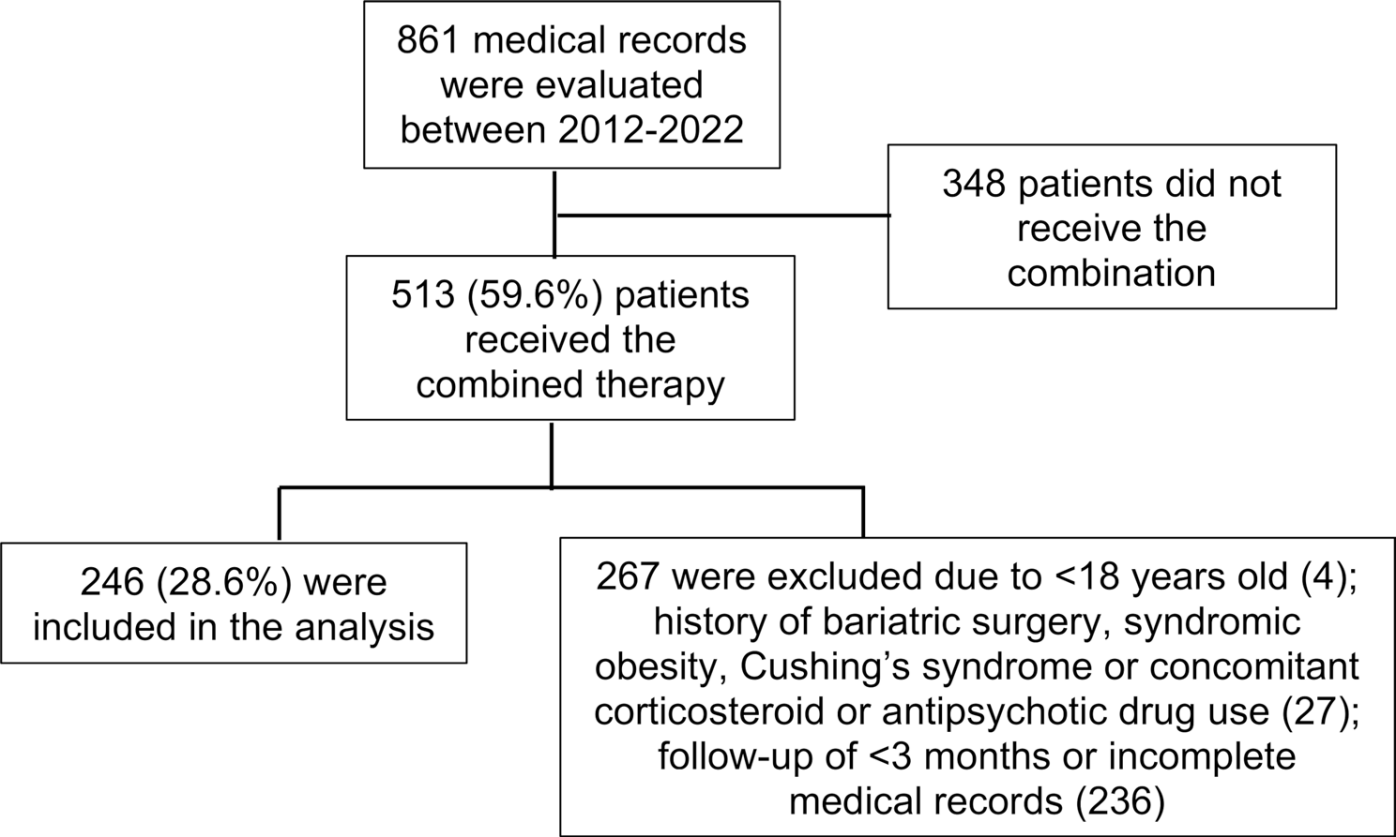
– Enrollment flowchart

Patients were excluded if they met any of the following:

- On-label contraindication to the drugs (i.e. established cardiovascular disease, *≥* 65 years, uncontrolled hypertension *≥* 145/90 mmHg, diabetes associated with another cardiovascular risk factor for sibutramine, personal history of angle-closure glaucoma for topiramate);
- Non-compliance with prescribed therapy or follow-up < 3 months;
- Incomplete medical record;
- Pregnancy or breastfeeding;
- Concomitant medications affecting body weight, such as antipsychotics, corticosteroids or other antiobesity drug;
- Previous surgical procedure for weight loss;
- Presence of a clinical condition related to severe obesity, such as Cushing’s syndrome or a syndromic form of obesity. Fig. 1 summarizes the patient selection process.

All participants received hypocaloric diet orientation and were encouraged to participate in physical activities (150 min or more per week). The hypocaloric diet was empirically orientated to a maximum of 1200–1600 kcal per day. For participants who had performed bioelectrical impedance analysis (InBody 770), a 500-kcal deficit from basal metabolic rate was prescribed. The patients also had regular follow-up with a multidisciplinary team composed of endocrinologists and nutritionists. The frequency of visits was individualized. Patients could also be scheduled for psychiatric and psychologic appointments if clinically indicated. Since topiramate is teratogenic, all women of childbearing age were orientated on contraceptive methods.

Regarding the prescribed dose, sibutramine was always started with 10 mg/d and topiramate with 25 mg/d. In patients with good tolerability and insufficient weight loss, the doses could be increased at the physician’s judgment, for up to 20 mg/d of sibutramine [12] and 300 mg/d of topiramate [13]. The dose escalation regimen was done within the first 3 months of combined therapy. After this period, the dose used was maintained stable and the information related to the maximum daily dose tolerated was recorded.

All data were obtained from medical records between 2012 and 2022. Demographic characteristics, medical history of comorbidities, self-reported side effects, and cardiometabolic biomarkers (laboratory test exams and blood pressure) were collected.

Continuous variables were summarized as mean ± standard deviation, while categorical variables were reported as count and percentage. Normality was assessed using the Shapiro-Wilk test. Differences between groups were evaluated with the Wilcoxon test for two-period comparisons and the Friedman test with Bonferroni correction for multiple timepoints. The *p*-value < 0.05 was considered statistically significant. Statistical analysis of the data was performed using Statistical Package for Social Science (SPSS) version 25.0. The study was approved by the local Ethics Committee.

## Results

A total of 246 patients were evaluated. Most of the population were young women with a mean BMI close to class III obesity (Table 1). The mean time of follow-up was 25.3 months.

**Table 1:** – Demographic, clinical and laboratory characteristics of the patients

Mean age, years	42.8 *±* 12.7
Female sex, n (%)	212 (86.2)
Baseline BMI, kg/m^2^	39.7 *±* 6.3
Baseline weight, kg	104.2 *±* 20.1
Abdominal circumference, cm	114.6 *±* 15.4
Fasting glucose, mg/dL	100.6 *±* 30.9
Total cholesterol, mg/dL	184.4 *±* 31.8
HDL, mg/dL	51.2 *±* 15.7
LDL, mg/dL	108.2 *±* 26.3
Triglycerides, mg/dL	133.8 *±* 102.2
Systolic blood pressure, mmHg	122.9 *±* 13.1
Diastolic blood pressure, mmHg	79.8 *±* 10.5
Comorbidities, n (%)	
Hypertension	128 (52)
Type 2 diabetes	77 (31.3)
Dyslipidemia	74 (30)

The most common dosage combination was 10 mg/d of sibutramine plus 100 mg mg/d of topiramate, in 64 (26%) participants (Table 2). Topiramate dosing had more variability, with 36 (14.6%) using 50 mg/d, 81 (32.9%) 100 mg/d and 38 (15.4%) 200 mg/d. The mean dose of topiramate was 119.7 ± 54.7 mg/d. On the other hand, 196 (79.7%) patients used the same maximum daily dose of 10 mg/d for sibutramine, with a mean dose of 11 *±* 2.1 mg/d. Only two patients tolerated 300 mg/d of topiramate and just one 20 mg/d of sibutramine.

**Table 2:** – Doses prescribed of sibutramine and topiramate

		Maximum daily dose of sibutramine		
		10 mg	15 mg	20 mg
Maximum daily dose of topiramate	25 mg	2 (0.8%)	1 (0.4%)	
	50 mg	34 (13.8%)	2 (0.8%)	-
	75 mg	24 (9.7%)	3 (1.2%)	-
	100 mg	64 (26%)	17 (6.9%)	-
	125 mg	6 (2.4%)	3 (1.2%)	-
	150 mg	35 (14.2%)	5 (2%)	1 (0.4%)
	175 mg	2 (0.8%)	2 (0.8%)	-
	200 mg	24 (9.7%)	14 (5.7%)	-
	250 mg	3 (1.2%)	2 (0.8%)	-
	300 mg	2 (0.8%)	-	-

Of note, some participants received monotherapy with either topiramate or sibutramine before the combination (full data not shown), therefore reducing the mean weight at the beginning of the association to 100.1 *±* 20.9 kg (Table 3). However, for comparison purpose, the weight used was the baseline before the initiation of any antiobesity drug. Significant weight loss was observed at 3 months (mean weight 96.8 ± 20.7 kg, *p* < 0.001), with a plateau phase beyond this period. The mean total weight loss at 36 months was − 10.2 ± 9.8 kg.

The only metabolic markers that improved during follow-up were HDL and diastolic blood pressure, although fasting glucose, total cholesterol, LDL and triglycerides had a non-significant reduction (Table 3).

**Table 3:** – Comparison between total weight and other risk factors during follow-up

Variables	Baseline	Association prescribed	3 m	6 m	12 m	24 m	36 m	*p*
Mean weight, kg	104.2 *±* 20.1	100.1 *±* 20.9ª	96.8 *±* 20.7ª	95.1 *±* 21.4	94.3 *±* 20.2	92.4 *±* 19.2	93.7 *±* 21.8	< 0.001*
Fasting glucose, mg/dL	100.6 *±* 30.9	-	-	-	98.7 *±* 28.5	-	-	0.053º
Total cholesterol, mg/dL	184.4 *±* 31.8	-	-	-	183.2 *±* 35.3	-	-	0.876º
HDL, mg/dL	51.2 *±* 15.7	-	-	-	53.1 *±* 15.1	-	-	**0.024**º
LDL, mg/dL	108.2 *±* 26.3	-	-	-	106.1 *±* 30.7	-	-	0.336º
Triglycerides, mg/dL	133.8 *±* 102.2	-	-	-	129.8 *±* 87.6	-	-	0.514º
Systolic blood pressure, mmHg	122.9 *±* 13.1	-	-	-	122.6 *±* 15.7	-	-	0.936º
Diastolic blood pressure, mmHg	79.8 *±* 10.5	-	-	-	73.1 *±* 20.7	-	-	**0.01**º

ªStatistically significance in weight loss when compared to the previous weight assessment period

*Friedman test with Bonferroni correction

ºWilcoxon test

**Fig. 2:**
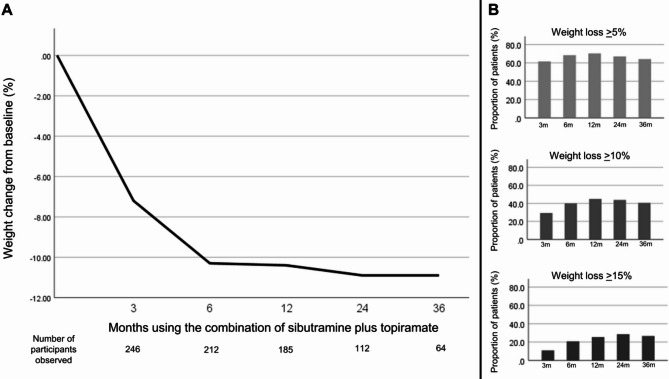
– Percentage of weight loss during follow-up

The mean percentage of weight loss (Fig. 2A) at 3 months was 7.2 *±* 6.5%, 10.3 *±* 11.6% at 6 months, 10.4 *±* 9.9% at 12 months, 10.9 *±* 12.1% at 24 months and 10.9 *±* 11.2% at 36 months, with no statistical difference between them. Evaluating only 3 months on the combination, 61.8% of patients achieved at least *≥* 5% of weight loss, 29.4% *≥*10% and 10.9% *≥*15%. This distribution was similar in the other assessed periods (Fig. 2B). At 36 months, 41/64 (64%) maintained at least *≥* 5% of weight loss, 26/64 (40.6%) *≥* 10% and 17/64 (26.5%) *≥* 15%.

A few individuals reported side effects, being the most common paresthesia (28%), probably related to the topiramate effect in carbonic anhydrase inhibition, and increased blood pressure (13%) and palpitation (8.9%), possibly correlated with sibutramine (Table 4). Discontinuation occurred in 60 patients (24.4%), with the primary reasons being uncontrolled hypertension *≥* 145/90 mmHg in 12 (4.9%), refractory paresthesia in 8 (3.2%) and tachycardia in 7 (2.8%).

**Table 4:** – Evaluation of main side effects and safety concerns

Complaint	*n* (%)
Paresthesia	69 (28)
Memory impairment	43 (17.5)
Bradyphrenia	33 (13.4)
Increased blood pressure*	32 (13)
Palpitation	22 (8.9)
Headache	9 (3.6)
Insomnia	8 (3.2)
Dizziness	8 (3.2)

*Increase of at least *≥* 10 mmHg in systolic or *≥* 5 mmHg in diastolic blood pressure compared to the baseline

## Discussion

The present pivotal study presents real-world evidence on the use of the sibutramine-topiramate combination for weight loss. These two drugs have low cost, are widely available in our country, and have extensive literature experience for security and tolerability when prescribed as monotherapy for obesity management​ [12–14].

In this study, the potency of weight loss reached was comparable to the reported in previous trials with phentermine plus topiramate [9]. This was highly expected, since although sibutramine is not a catecholaminergic drug as phentermine, it acts centrally, inhibiting the reuptake of both serotonin and norepinephrine, reducing the appetite and enhancing indirectly caloric expenditure [15]. To date, the combination phentermine-topiramate is one of the most efficacious oral medications approved on label for the treatment of obesity, with a mean weight loss surpassing the combination of bupropion plus naltrexone [16] and as potent as 3 mg subcutaneous liraglutide [17].

Sibutramine was withdrawn from the market in several countries due to cardiovascular safety after the publication of the SCOUT trial [18]. When analyzing all patients included, the mean weight loss with sibutramine was very poor, about 4 kg (less than 5%), and the continuation of therapy in this scenario would only increase side effects such as hypertension and tachyarrhythmia, partially explaining its unfavorable cardiovascular outcomes. However, in a post hoc subanalysis of this same trial, in good responders who lost a significant amount of weight, sibutramine actually decreased cardiovascular deaths [19]. Big cohort trials later on also provided cardiovascular security in those with no cardiovascular disease [20], demonstrating that sibutramine’s marketing authorization may have been inappropriately withdrawn for healthy young patients with obesity.

Topiramate, as a monotherapy, is not approved by the FDA for obesity treatment, but has several studies reporting substantial weight loss [13, 21]. It is believed that it acts on the arcuate nucleus, modulating GABA and glutamate receptors in the lateral hypothalamus, inhibiting orexigenic pathways and lowering dopamine release in the nucleus accumbens, reducing addiction, binge and compulsive behavior [21, 22]. Therefore, while sibutramine acts mainly on the homeostatic control of food intake, topiramate acts on hedonic and reward centers, making this a synergic combination.

Weight loss, even in a small amount, is related to the improvement in cardiovascular risk factors, such as lipid levels, glucose metabolism and blood pressure [3]. There was a tendency for improvement in all biomarkers evaluated in the present article, but since this is a non-interventional study, it is not possible to exclude possible confounding factors, as many patients started treatment with statins, antihypertensives and antidiabetics throughout follow-up.

The majority of side effects presented are well-reported in the literature. Topiramate can be associated with brain fog and paresthesia due to its central action and slight inhibition of carbonic anhydrase [23], respectively. Sibutramine, due to its noradrenergic effect, can increase blood pressure and predispose to tachyarrhythmia [18]. The rate of discontinuation due to adverse effects encountered of ~ 25% was comparable to that observed with other oral combinations [9, 16].

Probably the future of obesity treatment will be accompanied by several changes as observed for other chronic conditions. Recently, as an example, the European Society of Cardiology [24] released a new guideline for hypertension treatment and stated that all patients should receive as first-line therapy a combination of two antihypertensive drugs. The reason is that the combination is more potent for lowering blood pressure than a monotherapy in its highest dose. This finding is also applicable for obesity management as seen here with the combination of sibutramine plus topiramate, but also for triple GLP-1/GIP/glucagon agonist [8] versus GLP-1 agonist alone [6, 17].

In fact, recent studies showed that combined GLP-1 analogs and bupropion plus naltrexone are a very effective treatment for weight loss even when the dose used is not maximal. Moreover, the addition of bupropion plus naltrexone achieved greater weight loss, including in patients who were initially non-responsive to GLP-1 analogs [25, 26].

Although a weight loss of 5% can have multiple health benefits [3], probably at a later date, with the emergence of potent drugs, patients with obesity will start having a target BMI, similarly as seen in other chronic conditions that have predefined targets of LDL, HbA1c and blood pressure. The presence of multiple subtypes of antiobesity drugs targeting different mechanisms of action should aid in avoiding therapeutic inertia and pursuing this goal [11], if indicated by future guidelines. Therefore, even in the actual context, sibutramine plus topiramate could still be used for enhancing weight loss even in people using more potent drugs. However, its main focus for prescription should be on vulnerable patients with obesity due to its low cost [27]. This is of great importance since sibutramine is mainly available in third-world countries.

This study has some limitations. The data was derived from a single center with a variable dose used in the combination and the majority of the participants were healthy young women. The results might not be extrapolated for other populations. In addition, due to its retrospective design and the lack of a control group, it can only be assumed that the combination is superior to other treatment modalities. The inclusion of patients who used at least 3 months of the combination could also incur bias, potentially excluding non-compliant participants and possible early non-responders, although reflecting real life. Besides that, the large sample size included should be noticed as a strength.

The combination of sibutramine and topiramate may continue to play a role in obesity management, particularly in resource-limited settings. However, the development of newer pharmacotherapies targeting diverse pathways is likely to redefine treatment paradigms. Future research should focus on identifying optimal combinations and exploring personalized approaches to achieve weight loss goals.

## Conclusion

In a real-world setting, the combination of sibutramine and topiramate was associated with clinically meaningful weight loss. The results underscore its potential utility, particularly for patients in resource-limited scenarios, where cost-effective options are essential. The suggestive dosage of this combination should be 10 mg of sibutramine and 100 mg of topiramate per day, which was the most commonly prescribed. To the authors’ best knowledge, this is the first study specifically addressing combined sibutramine and topiramate therapy for obesity management, showing a long-term security profile in well-selected candidates for prescription.

## Data Availability

No datasets were generated or analysed during the current study.
